# Effects of Keluoxin capsule combined with losartan potassium on diabetic kidney disease: study protocol for a randomized double-blind placebo-controlled multicenter clinical trial

**DOI:** 10.1186/s13063-020-04852-8

**Published:** 2020-11-23

**Authors:** Rui Wu, Fan Wei, Lianlian Qu, Litao Bai, Jun Li, Fei Li, Weitian Yan, Qiuhong Wang, Junping Wei

**Affiliations:** 1 Department of Endocrinology, Guang’anmen Hospital, China Academy of Chinese Medical Sciences, Xichen District, Beijing, 100053 China; 2 Dermatological Department, Guang’anmen Hospital, China Academy of Chinese Medical Sciences, Xichen District, Beijing, 100053 China; 3 Department of Endocrinology, Penglai Traditional Chinese Medicine Hospital, Penglai, 265600 Shandong China; 4 Department of Integrated Chinese and Western Medicine, The Second Affiliated Hospital of Chongqing Medical University, Yuzhong District, Chongqing, 400010 China

**Keywords:** Chinese medicine, Diabetic kidney disease, Protocol, Randomized controlled trial

## Abstract

**Background:**

Diabetic kidney disease (DKD) is one of the most important microvascular complications of diabetes, and its prevalence has increased dramatically in the past few decades. DKD is responsible for considerable morbidity and mortality of patients with diabetes. Keluoxin capsule (KLX) is a Chinese patent medicine that has been used in the clinic to control DKD for years. Previous studies have shown that KLX appears to reduce proteinuria, but the study protocols as well as the primary outcome need to be improved. Thus, we aim to evaluate whether losartan potassium combined with KLX is more effective than losartan potassium in DKD treatment and to provide validated evidence for the application of KLX in the treatment of DKD.

**Methods:**

We will conduct a randomized double-blind placebo-controlled multicenter clinical trial. A total of 252 participants diagnosed with DKD recruited from 18 institutions will be randomly allocated to either a losartan potassium plus KLX (*n* = 126) or a losartan potassium plus placebo group (*n* = 126). The participants will be administered KLX or placebo in addition to losartan potassium for 24 weeks. The primary outcome measure will be the decline in estimated glomerular filtration rate (eGFR) (ml/min/1.73 m^2^/year) from baseline within 24 weeks, and the secondary outcomes will be the incidence of serum creatinine doubling, the incidence of end-stage renal disease (ESRD), the proportion of subjects with a progressive decline in eGFR > 30%, the percent change in 24 h urinary total protein (UTP), the change in the urinary albumin/creatinine ratio (UACR), and the total effective rate of the traditional Chinese medicine (TCM) syndrome scale scores. Comparison of the differences in the variables between groups will be performed according to the data revealed by independent *t* tests, chi-squared tests, Fisher’s exact tests, or Wilcoxon’s tests. All statistical tests will be two-sided, and significance will be considered for *p* values < 0.05.

**Discussion:**

This study will be the first randomized clinical trial to evaluate the efficacy and safety of KLX versus the placebo for the treatment of patients with DKD. The outcome of this trial will provide a basis for prescribing KLX to patients with DKD.

**Trial registration:**

Chinese Clinical Trial Registry (www.chictr.org.cn) ChiCTR1900021113. Registered on January 29, 2019.

## Background

Diabetic kidney disease (DKD), a chronic progressive disorder that is characterized by proteinuria and an irreversible decline in renal function, ultimately leads to end-stage renal disease (ESRD) in approximately 50% of patients in developed countries [1, 2]. DKD is one of the most important microvascular complications of diabetes, and its prevalence has increased dramatically worldwide in the past few decades [3, 4]. DKD accounts for approximately 20% of new dialysis prescriptions [5], and the high medical cost of renal dialysis treatment has created an urgent need for additional therapeutic strategies for DKD [6]. Comprehensive measures such as hypoglycemic and antihypertensive treatments have demonstrated beneficial effects in preventing the development of DKD, especially when used at the stage of microalbuminuria, and angiotensin-converting enzyme inhibitors (ACEIs) and angiotensin receptor II blockers (ARBs) reduce the risk of progression of DKD [7–9]. Losartan potassium, a classic angiotensin receptor blocker, is helpful in protecting the kidneys from damage in DKD [10]. In addition, this drug was recommended on the list of essential medicines of World Health Organization, which included the most effective and safe medications that experts consider necessary in a healthcare system. Treatment with losartan potassium may preserve some features of kidney structure and microalbuminuria due to its action on attenuating kidney fibrosis [11, 12]. However, combinations of traditional Chinese medicine (TCM) and Western medicine have advantages over Western medicine in delaying the progression of CKD and improving quality of life.

Keluoxin capsule (KLX) is a Chinese patent drug developed by the Chengdu Kanghong Pharmaceutical Co., Ltd. and Guang’anmen Hospital of the China Academy of Chinese Medical Sciences. KLX consists of six natural herbs: *Astragalus membranaceus*, *Ligustrum lucidum*, leech, rhubarb, Radix Pseudostellariae, and the fruit of the Chinese wolfberry (https://www.cnkh.com/product/17156.htm). KLX is used for DKD in patients with syndromes of qi and yin deficiency and blood stasis because it nourishes qi and yin, promotes blood circulation, and removes blood stasis. KLX was once named Tangshenping or Tangweikang [13, 14]. Research on KLX for DKD is included in the national Ninth Five-Year Plan project.

KLX was placed on the market in 2009 (the approval number is National Medicine Standard Z20090035) and is considered a B category drug by Chinese medical insurance. Previous studies have shown that KLX can regulate glucose and lipid metabolism, improve renal hemodynamic parameters, and protect against pathological damage in diabetic rats [13]. KLX can also reduce high glomerular perfusion and filtration rates and thus delay the processes of glomerular fibrosis and hardening in diabetic nephropathy rat models [15]. In addition, KLX was approved for clinical trials by the State Food and Drug Administration in 2003. The results of the trials showed that KLX is effective in controlling 24 h urinary total protein (UTP) levels, urinary albumin (ALB)/creatinine ratios (UACRs), and TCM syndrome scale scores [13, 16]. Moreover, both acute toxicity tests and long-term toxicity tests have verified the safety KLX in the treatment of DKD.

However, there is a lack of validated evidence regarding the effectiveness of KLX in DKD. Previously, the efficacy evaluation of KLX is focused on urine protein, but the influence of KLX on kidney progression of DKD, which is assessed by a change from baseline in the eGFR, has not been previously assessed. Therefore, in the proposed study, we will conduct a randomized double-blind placebo-controlled multicenter clinical trial to evaluate whether KLX combined with losartan potassium tablets is more effective in delaying the progression of DKD than losartan potassium tablets based on the pathophysiological mechanism of the development of DKD.

## Methods/design

### Study objectives

The objective of this trial is to evaluate whether the effect of losartan potassium combined with KLX is superior to that of losartan potassium in DKD treatment and to obtain validated evidence for the application of KLX in the treatment of DKD.

### Study design

**Fig. 1.**
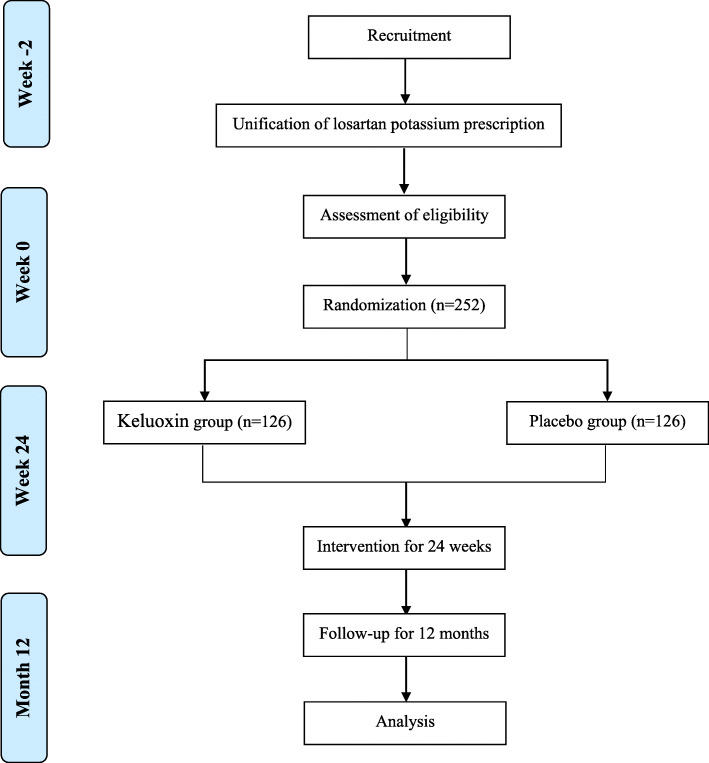
Trial flowchart

This will be a double-blind randomized controlled trial to investigate the efficacy of KLX combined with losartan potassium compared with a placebo. The study will be conducted in eighteen centers in China: Guang’anmen Hospital of the China Academy of Chinese Medical Sciences, Dongzhimen Hospital of Beijing University of Chinese Medicine, the First Affiliated Hospital of Chengdu Medical College, Mianyang Central Hospital, the Affiliated Hospital of Chengdu University of Traditional Chinese Medicine, Dazhou Central Hospital, Dezhou People’s Hospital, Linfen Central Hospital, the Affiliated Hospital of Liaoning University of Traditional Chinese Medicine, the First People’s Hospital of Luoyang, Shenzhen Hospital of Southern Medical University, Shanghai Shuguang Hospital, Yueyang Hospital of Integrated Traditional Chinese and Western Medicine, Shanghai University of Traditional Chinese Medicine, the Second Affiliated Hospital of Wannan Medical College, Nanyang City Central Hospital, the First Affiliated Hospital of Henan University of Chinese Medicine, Xinxiang Central Hospital, and Heze Municipal Hospital. Patients with DKD who want to participate in the study will undergo a standardized baseline evaluation before treatment including detailed history evaluation, physical examination, and laboratory testing. The enrolled participants will be randomly allocated to the losartan potassium plus KLX treatment or losartan potassium plus placebo control groups. The participants will be administered KLX or placebo in addition to losartan potassium for 24 weeks. Data will be collected during three visits to assess the efficacy of KLX and will be recorded on case report forms (CRFs). A flowchart of the study design is shown in Fig. 1, and the time points of assessment are shown in Table 1. The Standard Protocol Items: Recommendations for Interventional Trials (SPIRIT) guidelines [17] were followed during the development of the protocol of this study (Additional file 2).

**Table 1:**
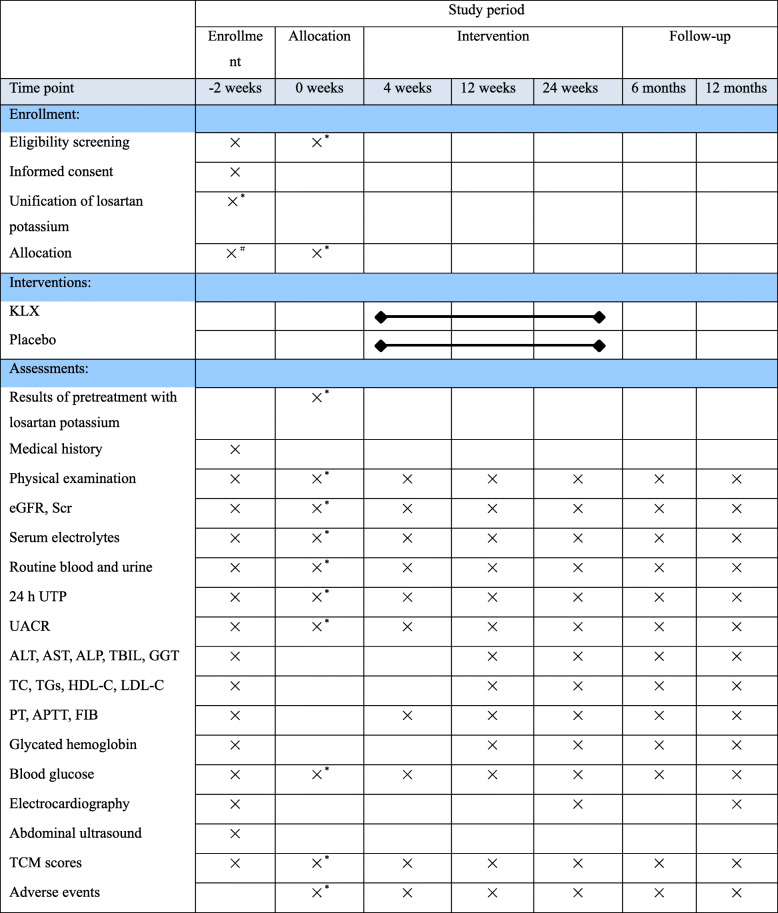
Schedule of enrollment, intervention, and assessment

*Abbreviations*: *24 h UTP* 24 h urinary total protein, *UACR* urinary albumin/creatinine ratio, *eGFR* estimated glomerular filtration rate, *Scr* serum creatinine, *PT* prothrombin time, *APTT* activated partial thromboplastin time, *FIB* fibrinogen *ESRD* end-stage renal disease, *ALT* alanine transaminase, *AST* aspartate aminotransferase, *GGT* gamma-glutamyl transpeptidase, *TC* total cholesterol, *TGs* triglycerides, *LDL-C* low-density lipoprotein cholesterol, *HDL-C* high-density lipoprotein cholesterol, *TCM* traditional Chinese medicine

“×^*^” only for subjects that undergo losartan potassium unification (50 mg per day). “×^#^” only for subjects that take losartan potassium at 50 mg per day before enrollment

### Study settings and recruitment

Assuming a 20% dropout rate, a total of 252 patients will be recruited from the eighteen centers mentioned above using posters, the hospitals’ websites, and networks from December 2019 to December 2020. Research assistants will manage the recruitment, and endocrinologists will diagnose the participants. On the consent form, participants will be asked for permission to apply their data in case of withdrawal. Participants will also be asked to agree to share the research data with people from the hospitals participating in the study or from regulatory authorities where relevant. Biological specimens are not stored in this study. The trial adopted clinical trial insurance, and affected participants will receive appropriate coverage if serious harm occurs following the trial.

### Participants

Subjects will be deemed eligible participants if they meet all of the listed inclusion criteria and none of the listed exclusion criteria.

#### Inclusion criteria

1. Diagnoses of type 2 diabetes based on the American Diabetes Association (ADA) criteria [18] and DKD defined by *National Kidney Foundation Kidney Disease Outcomes Quality Initiative (NKF K-DOQI) guidelines* [19]. Participants who meet any of the following criteria can be considered as DKD: a, large amounts of albuminuria; b, diabetic retinopathy with chronic kidney disease at any stage; and c, microalbuminuria combined with a greater than 10-year course of type 1 diabetes.
2. Diagnoses of TCM syndromes of qi and yin deficiency and blood stasis were constructed based on Diagnosis and Treatment of Diabetes and Its Complications with Integrated Chinese and Western Medicine as follows: Primary signs/symptoms include dry mouth and throat, and fatigue. Secondary signs/symptoms include a propensity to be hungry and eat more; shortness of breath; heat sensation in chest, palms, and soles; fixed pain in the chest, lower back, or limbs; and numbness of the limbs. Participants who suffered from the primary signs/symptoms and at least two of the secondary signs/symptoms combined with the tongue manifestation are considered to exhibit syndromes of qi and yin deficiency and blood stasis.
3. A UACR ≥ 30 mg/g.
4. An estimated glomerular filtration rate (eGFR, according to the Chronic Kidney Disease Epidemiology Collaboration (CKD-EPI) equation) ≥ 30 ml/min/1.73 m^2^ and ≤ 59 ml/min/1.73 m^2^.
5. A fasting blood glucose level < 13.9 mmol/l and/or a 2-h postprandial blood glucose level < 16.6 mmol/l.
6. A glycated hemoglobin (hemoglobin A1c, HbA1c) percentage ≤ 10%.
7. An age between 35 and 75 years, male or female.
8. Voluntary participation in this clinical study and provision of written informed consent.

#### Exclusion criteria

1. A diagnosis of type 1 diabetes.
2. A lack of diagnosed diabetic retinopathy.
3. A history of simple renal hematuria or proteinuria with hematuria, sudden onset of edema and mass proteinuria without abnormal renal function, significant renal tubular dysfunction, coexisting renal tubular abnormalities, primary glomerulonephritis or secondary nephritis except DKD, or acute or chronic infection of the urinary system.
4. A hemoglobin (HGB) concentration < 90 g/l.
5. An ALB concentration < 30 g/l.
6. A diagnosis of renal artery stenosis.
7. A serum potassium concentration > 5.5 mmol/l.
8. A serum creatinine (Scr) concentration ≥ 3 mg/dl (256 μmol/l).
9. A history of severe cardiovascular or cerebrovascular disease within 3 months before screening.
10. Severe systemic primary disease or dysfunction.
11. Severe liver dysfunction or ALT or AST levels higher than 2.5 times the normal range.
12. Hypertension treated with more than 3 types of antihypertensive drugs, blood pressure that is poorly controlled after administration of drugs with systolic blood pressure (SBP) ≥ 140 mmHg or diastolic blood pressure (DBP) ≥ 90 mmHg, or hypotension with SBP ≤ 90 mmHg or DBP ≤ 60 mmHg (resting position).
13. Chronic (for more than 3 weeks) or recurrent diarrhea (where diarrhea is defined as elimination of increased volumes of thin fecal matter with increased frequency (> 3 times/day)).
14. A history of active bleeding in the 3 months before screening.
15. Coagulation disorders.
16. Drug allergies or allergies to KLX or the active ingredient in losartan potassium.
17. A history of alcohol or drug abuse or psychiatric disorders.
18. Pregnancy, lactation, or plans to become pregnant during the trial.
19. A history of enrollment in other clinical studies in the past 3 months.
20. Other circumstances under which the investigators considered it inappropriate to participate in this clinical study.

### Randomization, allocation concealment, and blinding

The participants will have an individual trial identification number and be randomly assigned to either the losartan potassium plus KLX group or the losartan potassium plus placebo group in a 1:1 ratio. Randomization will be performed using Statistics Analysis System (SAS) software by an independent statistical agency. The Data Management Center of Guang’anmen Hospital will be responsible for drug blinding and randomization concealment. The statistician will not participate in the randomization and will analyze the collected data without having access to allocation information. Research assistants will enroll patients at the 18 participating medical centers sequentially on the basis of screening order. Both the participants and the investigators will be kept blinded to the allocations until the trial is completed. In case of a severe adverse event, an administrator at the Data Management Center of Guang’anmen Hospital will unblind the patient information as an emergency measure and provide appropriate treatment.

### Intervention

All investigators will be trained before the start of the study according to an investigators’ brochure. The enrolled patients with DKD will be asked to take losartan potassium tablets (50 mg per day) as the basic treatment and will also be administered KLX or placebo. The subjects in the treatment group will take four capsules of KLX orally thrice a day for 24 weeks. The participants in the control group will take the same amount of placebo for 24 weeks. Data from all the participants will be collected in four periods: on day 0 (V2), at 4 weeks ± 3 days (V3), at 12 weeks ± 5 days (V4), and at 24 weeks ± 5 days (V5).

KLX consists of six natural herbs: *Astragalus membranaceus*, *Ligustrum lucidum*, leech, rhubarb, Radix Pseudostellariae, and the fruit of the Chinese wolfberry. The placebo is identical in appearance and properties to KLX capsule without any active ingredient. Both KLX and the placebo will be manufactured by Chengdu Kanghong Pharmaceutical Co., Ltd., at a dose of 500 mg. The placebo, which will be identical in appearance and properties to KLX, will contain no active ingredients. The placebo will be verified by the quality control department and will meet the standards of placebo preparation. The interventions should stop for a given participant if the creatinine doubles or ESRD is detected. The participants will be asked to refrain from taking any other treatment for diabetic nephropathy, including ACEI or ARB drugs other than losartan potassium tablets and calcium hydroxybenzenesulfonate as well as Chinese medicine that affect evaluation of qi and yin deficiency and blood stasis syndrome during the trial.

### Outcome measures

#### Primary outcomes

The primary endpoint is the decline in the eGFR (ml/min/1.73 m^2^/year).

#### Secondary outcomes

The secondary outcomes will include the incidence of Scr doubling, the incidence of ESRD, the proportion of subjects with a progressive decline in eGFR of > 30% from baseline, the percent change in 24-h UTP, the change in UACR from baseline, and the total effective rate of TCM syndrome scale scores at every visit and follow-up point, including the 4th, 12th, and 24th weeks as well as the 6th and 12th months.

#### Safety assessments

To assess the safety of KLX treatment in patients with DKD, vital signs and some laboratory parameters will be measured and electrocardiography will be performed during the intervention period of the trial. In detail, the vital signs will include body temperature, blood pressure, respiratory rate, and pulse rate. The biological indicators we will monitor will include routine blood indices (red blood cell count (RBCs), hemoglobin(HGB), white blood cell count (WBCs), and platelet count (PLTs)), routine urine and urine sediment parameters (assessed under microscopy), 24-h UTP levels, UACRs, liver function indices (alanine aminotransferase (ALT), aspartate aminotransferase (AST), alkaline phosphatase (ALP), total bilirubin (TBIL), and γ-glutamyl transferase (GGT)), renal function indices (eGFR and Scr), serum electrolytes (K^+^, Na^+^, and Cl^−^), blood lipid profile indices (total cholesterol (TC), triglycerides (TGs), high-density lipoprotein cholesterol (HDL-C), and low-density lipoprotein cholesterol (LDL-C)), coagulatory function indices (prothrombin time (PT), activated partial thromboplastin time (APTT), and fibrinogen (FIB)), HbA1c levels, and fasting blood glucose levels. To reduce risk, vital signs and some laboratory parameters of the participants will be monitored during the intervention period of the trial. Clinical experts will evaluate the participants’ condition at every visit. Moreover, detailed information about any adverse events will be recorded, including the severity, rate of incidence, and correlation with the treatment. Adverse events will be monitored and treated until properly resolved.

### Compliance

The investigators are responsible for informing and supervising the participants to take the products strictly according to regulations. The details about medication should be reported and recorded at every visit in the case report forms. Those with drug doses less than 80% or greater than 120% of the prescribed amount will be withdrawn from the trial and considered as noncompliance. Moreover, the specific withdrawal reasons should be recorded in CRFs as well.

### Follow-up

All included participants will be re-evaluated at 6 and 12 months through phone calls or as outpatients.

### Statistics

#### Sample size

This study will be a trial of a new therapeutic regimen of KLX treatment. The sample size was calculated using software PASS based on a similar study on a Chinese herbal medicinal supplement conducted previously [20, 21]. According to the results of our previous trial, the mean decline of eGFR in the expected population was assumed to be 1.38 ± 3.21 and 2.61 ± 3.16 ml/min/1.73 m^2^/half a year in losartan potassium plus KLX versus losartan potassium plus placebo group, respectively. Therefore, we estimated that with a sample size of 105 patients assigned to each group (ratio 1:1) would be sufficient to achieve 90% power in detecting the differences of the primary endpoint based on a two-sided level of significance of 5%. Allowing for a 20% withdrawal rate, we plan to include 126 patients in this trial.

#### Data analyses

The independent researchers performing the biochemical tests, assessing the clinical outcomes, and analyzing the data will be kept blinded to the patients’ identities and grouping.

Continuous indicators are described as means, standard deviation, medians, and interquartile ranges, while classification variables are reported as absolute values and percentages. The following aspects should be analyzed at the end of the trial: the actual number of participants in the two groups, dropped and excluded cases, baseline characteristics, compliance, efficacy, and safety. To insure the comparability between groups, baseline comparisons, including the indicators of demographic characteristics, clinical characteristics, biochemical variables, medical history, and medication history (Tables 2 and 3), were performed according to the data features by independent *t* test, chi-squared test, or Fisher’s exact test.

**Table 2:** List of baseline characteristics of participants

Category	Items
Demographic characteristics	Age
	Sex
Clinical characteristics	Known duration of diabetes
	CKD stage
	Body mass index (height and weight)
	Blood pressure
Biochemical variables	eGFR, Scr
	UACR, 24 h UTP
	TC, TG, HDL-C, LDL-C
	ALT, AST, ALP, TBIL, GGT
	PT, APTT, FIB
	Blood glucose
	Glycated hemoglobin
Medical history	Hypertension
	Hyperlipidemia
	Coronary heart disease
	Congestive heart disease
	Stroke
	Peripheral vascular disease
	Others
Medication history	Drug name
	Drug dose
	Beginning drug use date
TCM evaluation scores	Details are listed in Table 3

**Table 3:** Traditional Chinese medicine (TCM) symptom assessment survey and evaluation standard

Signs/symptoms		Score				Scores
		Normal: 0	Mild: 2	Moderate: 4	Severe: 6	
Primary signs/symptoms	Dry mouth and throat	None	Decreased saliva production	Moderate thirst relieved by drinking fluids	Severe thirst with constant need to drink fluids	
	Fatigue	None	Cannot sustain heavy work	Can manage mild intensity work	Can only perform daily activities	
Secondary signs/symptoms	Prone to be hungry and eat more	None	Easy to be hungry	Easy to be hungry and unbearable hunger	Unbearable hunger and hypoglycemia	
	Shortness of breath	None	Shortness of breath after mild activity	Shortness of breath after moderate activity	Unable to talk or catch breath even without activity	
	Heat sensation in chest, palms, and soles	None	Heat sensation in palms and soles at night	Moderate heat sensation in chest, palms and soles	Severe heat sensation in chest, palms, and soles and desire to expose body	
	Fixed pain in the chest, lower back, or limbs	None	Occasional and relieved spontaneously within half an hour	Less than three hours a day, relieved by common drugs	Continuously fixed pain, relieved only by painkillers	
	Numbness of the limbs	None	Tips of hands and feet	Hands and feet	Limbs	
Tongue and pulse	Tongue	Dark red tongue with less fluid coating; others (description):				
	Pulse	Weak and thin pulse; others (description):				

Manifestation of tongue and pulse is only for reference without being calculated in the scores

Evaluation standard of total effective rate of TCM syndrome scale score

(1) Clinical recovery: the clinical symptoms and signs disappear or basically disappear, and syndrome scores are reduced by ≥ 95%

(2) Significantly effective: the clinical symptoms and signs are significantly improved, and syndrome scores are reduced by ≥ 70%

(3) Effective: the clinical symptoms and signs are improved, and syndrome scores are reduced by ≥ 30%

(4) Invalid: the clinical symptoms and signs are not improved, or even worsened, and syndrome scores are reduced by less than 30%

The calculation formula (Nimodipine method) is as follows: [(scores before treatment − scores after treatment) ÷ scores before treatment] × 100%

Total effective rate = (clinical recovery + significantly effective + effective) number of cases/total number of cases × 100%

The total effective rate of the KLX group and the control group after treatment is calculated and compared

Full Analysis Set (FAS), Per Protocol Set (PPS), and Safety Set (SS) are to be used for data analysis. According to intention-to-treat principle, FAS is defined as analysis of all cases who met all the inclusion criteria and none of the exclusion criteria, were randomized, and had at least one dose of the study drug. It included all enrolled and dropout cases except excluding participants. Multiple imputation analysis will be carried to explain the effect of missing data. Missing data of the primary outcomes will be carried forward with the principle of the last observation carried forward (LOCF). PPS includes the data from the subjects who follow the test protocol strictly, have good compliance, do not take the prohibited drugs during the test period, and complete the CRF form. SS refers to the participants who have made at least one visit and actual data recorded for safety indicators.

To evaluate the effect of KLX, data were analyzed primarily using an intention-to-treat principle, and per-protocol analysis served as a supplement. For the primary outcomes, we will calculate the changes of eGFR from baseline. To compare the decline in eGFR between two groups, repeated-measures analysis of covariance model will be performed using baseline eGFR as covariates. Least-square means with SEMs and two-sided 95% CIs for differences between groups were estimated. For secondary outcomes, statistical description and comparison between groups will be performed according to whether the variables are continuous or categorical data. To be specific, the difference in classification indicators among secondary outcomes, including the incidence of Scr doubling, the incidence of ESRD, and the proportion of subjects with a progressive decline in eGFR of > 30% from baseline within 24 weeks, between groups will be compared using chi-squared or Fisher’s exact test. While the difference in the total effective rate of TCM syndrome scale scores between the two groups will be tested as rank data using Wilcoxon’s test or CMH test. Comparison of the continuous variables, such as the percent change in 24-h UTP and the change in UACR, will be performed applying independent *t* tests (or the Mann-Whitney *U* test). Safety evaluation is composed of comparison of parameters listed above as well as adverse events evaluation. Besides the detailed description, reason, and explanation of the adverse events, the chi-squared test or Fisher’s exact test will be used to compare the incidence of AEs between the two groups.

There will be no interim analysis of the data because there are no anticipated problems that are detrimental to the participants. All the data will be analyzed using Statistical Package for the Social Sciences (SPSS) version 17.0 (SPSS, Inc., Chicago, IL) software. All statistical tests will be two-sided, and significance will be considered for *p* values < 0.05.

#### Data collection and management

All researchers in this study will be qualified physicians and will receive training in standard operating procedures for trial execution, biological sample collection, and handling. Based on the initial observation records, all center investigators will complete the CRFs in an accurate and timely manner. The sponsor and administrators of Guang’anmen Hospital will visit each center regularly to ensure the quality of data collection and to facilitate problem-solving. All data collected will be properly classified and saved/archived under confidential conditions and only accessed by researchers of the trial team. The Institute of Data Management Center of Guang’anmen Hospital is responsible for inspecting whether the trial is performed in compliance with the relevant regulations, GCP, and trial protocols.

#### Study management

The study is investigator sponsored and will be monitored and audited by the administrators of Guang’anmen Hospital to ensure that the trial to be conducted follows Good Clinical Practice principles. The administrators of Guang’anmen Hospital are also responsible for all aspects of local organization. The trial management committee (TMC) is composed of investigators from different research centers and administrators of Guang’anmen Hospital. The TMC will meet every 3 months during the study. The committee will provide guidance on day to day support for the trial, identify potential recruits, monitor the trial procedures, and report information to the trial steering committee (TSC). The TSC, which is independent from the TMC, will meet at least every 6 months to supervise the trial and offer guidance to ensure proper study conduct and progress. The Data Management Center and Ethics Committee of Guang’anmen Hospital will meet every 6 months to review conduct throughout the trial period. Supplementary Table 1 (S1) provides a description of the roles for the study groups that are responsible for trial management and monitoring.

#### Protocol amendments

If any amendments to the study are needed, the amendment will be agreed upon by the TMC and approved by the sponsor. Then, the administrators of Guang’anmen Hospital will notify the centers of the changes to the protocol. Any protocol changes will be fully documented using a breach report form, and the protocol will be subsequently updated in the clinical trial registry.

## Discussion

DKD is the largest contributor to ESRD worldwide and is the leading cause of morbidity and mortality in patients with diabetes [22]. Methods to control DKD are urgently needed due to the increasingly high incidence of diabetes worldwide. Treatment of DKD mainly focuses on glycemic control, blood pressure reduction, and renin-angiotensin-aldosterone system (RAAS) blockade [23, 24]. TCM plays considerable roles in symptom and quality of life improvement for DKD patients in China. Therapies integrating Chinese and Western medicine have shown some benefits in delaying the progress of DKD and improving the quality of life of patients [25]. KLX, an empirical prescription for DKD, has been used in the clinic for years. The proposed trial aims to examine the efficacy and safety of KLX in the treatment of mild DKD. The placebo was applied as the negative control to validate evidence for the application of KLX in the treatment of DKD. A prospective cohort design will be employed in the study, and 252 DKD patients will be treated as subjects.

Previous studies have demonstrated that KLX can reduce blood glucose levels and regulate lipid metabolism in DKD patients [14]. KLX also relieves glomerular endothelial cell damage, protects renal function, improves hemodynamics, and promotes renal microcirculation [13, 26]. However, there have been some limitations in previous clinical trials, such as lack of training for investigators, small sample sizes, and short observation periods. To ensure the quality of the trial, some specific measures will be taken in the proposed study. First, patients will be enrolled from 18 study centers to minimize bias, and all subjects will be screened strictly. Second, the investigators will be trained and asked to perform the trials according to the researcher’s manual. Third, the inclusion criteria will include eGFR and TCM syndrome scale scores. Both subjective and objective indicators have been selected for comprehensive assessment of efficacy. Fourth, a placebo similar in appearance and taste to KLX will be used. In addition, although the pathogenesis of DKD remains unclear, most scholars believe that it is closely related to abnormal renal hemodynamics and disorders of RAAS [27]. Therefore, ACEIs and ARBs are commonly used to treat DKD because both ACEIs and ARBs can antagonize the activity of RAAS and reduce glomerular sclerosis and proteinuria [9]. Hence, as a control variable, losartan potassium will be given to both the treatment and control groups from the screening time point until the end of intervention. Fifth, a third-party randomization scheme will be applied for allocation concealment and double blinding.

Some limitations in the current study should be noted. For example, the subjects are enrolled based on strict inclusion and exclusion criteria and paid close attention to by the investigators, which is quite different from the situation in real life. Therefore, the results of the trial may confine the application ranges of KXL. Moreover, it will be more complicated to manage and perform the trial in multiple centers.

Measurements are taken to eliminate potential bias. For example, samples collected from all the participating medical centers will be cold-chain transported to a single laboratory for processing, and the applied efficacy evaluation methods are strictly verified among the centers. Regardless, potential bias could be still generated from poor medication compliance, failure to attend an appointment within specified time, or cases that drop out.

This study will be the first randomized clinical trial to evaluate the efficacy and safety of KLX versus placebo for the treatment of patients with DKD. The outcome of this trial will provide a basis for prescribing KLX to patients with DKD.

## Trial status

The trial was carried out according to study protocol version 2.0 on December 6, 2019. Participant recruitment began in September 2019 and will finish in December 2020 as expected.

## Supplementary Information

**Additional file 1: Supplementary Table 1**. Description of the roles for the study groups.

**Additional file 2:.** Reporting checklist for protocol of a clinical trial.

**Additional file 3:.** Consent form.

## Data Availability

The study datasets will be available via the corresponding author upon reasonable request, and the results will be published in peer-reviewed journals.

## Abbreviations

DKD: Diabetic kidney disease
KLX: Keluoxin capsule
TCM: Traditional Chinese medicine
CRF: Case report form
eGFR: Estimated glomerular filtration rate
ESRD: End-stage renal disease
UTP: Urinary total protein
UACR: Urinary albumin/creatinine ratio
ACEI: Angiotensin-converting enzyme inhibitor
ARB: Angiotensin receptor II blocker
Scr: Serum creatinine
ALT: Alanine aminotransferase
AST: Aspartate aminotransferase
GGT: γ-Glutamyl transferase
ALP: Alkaline phosphatase
TBIL: Total bilirubin
TG: Triglyceride
TC: Total cholesterol
HDL-C: High-density lipoprotein cholesterol
LDL-C: Low-density lipoprotein cholesterol
PT: Prothrombin time
APTT: Activated partial thromboplastin time
FIB: Fibrinogen
HbA1c: Hemoglobin A1c
