# Supplementary material for: Effects of Keluoxin capsule combined with losartan potassium on diabetic kidney disease: study protocol for a randomized double-blind placebo-controlled multicenter clinical trial
Source: Trials. 2020 Nov 23;21:951. doi: 10.1186/s13063-020-04852-8 (PMC7682004; doi:10.1186/s13063-020-04852-8)
Supplement: Supplementary file 1 — Additional file 1: Supplementary Table 1. Description of the roles for the study groups. [file 13063_2020_4852_MOESM1_ESM.doc]

**Supplementary Table 1 - Description of the roles for the study groups**

| **Data category** | **Information** |
| --- | --- |
| Sponsor | Professor Junping Wei, Guang’anmen Hospital, China Academy of Chinese Medical Sciences, Xicheng District, Beijing 100053, China. Tel: (8610) 8800-1218. E-mail: weijunping@126.com.  The trial was financial funded supported by the project from Beijing Municipal Natural Science Foundation (NO. 7202172) which was in charge of Professor Junping Wei. |
| Funders | Chengdu Kanghong Pharmaceutical Co., Ltd. provided Keluoxin capsule and the placebo for free. Tel: 028-87516552. Email: 018401@cnkh.com. |
| Contact for public queries | Professor Junping Wei, Guang’anmen Hospital, China Academy of Chinese Medical Sciences, Xicheng District, Beijing 100053, China. Tel: (8610) 8800-1218. E-mail: weijunping@126.com. |
| Contact for scientific queries | Professor Junping Wei, Guang’anmen Hospital, China Academy of Chinese Medical Sciences, Xicheng District, Beijing 100053, China. Tel: (8610) 8800-1218. E-mail: weijunping@126.com. |
| Administrators of Guang’anmen Hospital for Coordinating | Professor Junping Wei, Guang’anmen Hospital, China Academy of Chinese Medical Sciences, Xicheng District, Beijing 100053, China. Tel: (8610) 8800-1218. E-mail: [weijunping@126.com](mailto:weijunping@126.com).  Dr. Rui Wu, Guang’anmen Hospital, China Academy of Chinese Medical Sciences, Xicheng District, Beijing 100053, China. Tel: (8610) 6028-3758. E-mail: [wurui19860420@163.com](mailto:wurui19860420@163.com).  Jun Li, Guang’anmen Hospital, China Academy of Chinese Medical Sciences, Xicheng District, Beijing 100053, China. Tel: 18811498007. E-mail: [18811498007@163.com](mailto:18811498007@163.com);  Fei Li, Guang’anmen Hospital, China Academy of Chinese Medical Sciences, Xicheng District, Beijing 100053, China. Tel: 15600790973. E-mail: mailto:845999010@qq.com;  Weitian Yan, Guang’anmen Hospital, China Academy of Chinese Medical Sciences, Xicheng District, Beijing 100053, China. Tel: 13001141072. E-mail: 604832711@qq.com;  Qiuhong Wang, Guang’anmen Hospital, China Academy of Chinese Medical Sciences, Xicheng District, Beijing 100053, China. Tel: (8610) 8800-1048. qiuhongfortune@126.com; |
| Trial Management Committee | Professor Junping Wei  Dr Lianlian Qu  Dr Litao Bai  Professor Xiaohui Yang  Professor Shaoqing Wang  Professor Shuhua Zhou  Professor Xiangming Fang  Professor Ming Chen  Professor Lin Liu  Professor Jianmin Wang  Professor Tianshu Gao  Professor Qiuwei Yang  Professor Xiaohua Guo  Professor Hao Lu  Professor Hongjie Yang  Professor Shaoping Tao  Professor Wenbo Jin  Professor Zhihai Feng  Professor Yanhong Zhou  Dr Fan Wei  Dr Jun Li  Dr Fei Li  Dr Weitian Yan |
| Trial Steering Committee | Professor Lan Lin (Consultant in Endocrine Medicine, Guang’anmen Hospital)  Professor Haibo Yin (Prof of internal diseases, Guang’anmen Hospital)  Professor Xinghua Feng (Prof of internal diseases, Guang’anmen Hospital)  Professor Wei Cao (Consultant in Renal Medicine, Guang’anmen Hospital)  Professor Jun Zhao (Prof of Psychology, Guang’anmen Hospital)  Dr. Ping Wu (Consultant in Research management, Guang’anmen Hospital)  Dr Lizhen Gu (Consultant in Pharmacology and Toxicology, Guang’anmen Hospital)  Dr Ruiying Shen (Consultant in Medical Ethics, Capital Medical University)  Dr Fei Li (Statistician, Guang’anmen Hospital) |
