# Supplementary material for: Effects of Keluoxin capsule combined with losartan potassium on diabetic kidney disease: study protocol for a randomized double-blind placebo-controlled multicenter clinical trial
Source: Trials. 2020 Nov 23;21:951. doi: 10.1186/s13063-020-04852-8 (PMC7682004; doi:10.1186/s13063-020-04852-8)
Supplement: Supplementary file 3 — Additional file 3:. Consent form. [file 13063_2020_4852_MOESM3_ESM.pdf]

渴络欣胶囊联合氯沙坦钾治疗糖尿病肾病（气阴两虚兼夹血瘀证）随机、双盲、安慰剂对照、多中心临床研究

Effects of Keluoxin Capsule Combined with Losartan Potassium on Diabetic Kidney Disease with Qi and Yin Deficiency and Blood Stasis Syndrome: Study Protocol for a Randomized Double-Blind Placebo-Controlled Multicenter Clinical Trial

受试者知情同意书·知情告知页

版本：2.0 版本日期：2019.12.06

Informed consent & Consent Form

Version: 2.0 Version Date: 2019.12.06

第一部分 受试者须知

Part 1 Notice for the participants

尊敬的受试者：

Dear participants:

我们将开展一项“渴络欣胶囊联合氯沙坦钾治疗糖尿病肾病（气阴两虚兼夹血瘀证）随机、双盲、安慰剂对照、多中心临床研究”，您的情况可能符合该项临床研究的入组条件，因此，我们邀请您参加该项研究。

在您决定是否同意参加该研究之前，请仔细阅读以下内容，它可以帮助您了解该项研究，为何要进行这项研究以及研究的程序和期限，参加研究后可能给您带来的益处、风险和不适。如果您愿意，您也可以和您的亲属、朋友一起讨论，或者请您的医生给予解释，协助您作出决定。

1. 为什么进行这项研究 ？

The purpose of this trial

渴络欣胶囊 (consist of Keluoxin capsule)由黄芪、女贞子、水蛭、大黄、太子参、枸杞子组成，具有益气养阴、活血化瘀的功效，用于糖尿病肾病属气阴两虚、兼夹血瘀者。

研究目的 (objectives): 为评价渴络欣胶囊联合氯沙坦钾延缓糖尿病肾病进展的疗效是否优于单用氯沙坦钾，拟开展随机、双盲、安慰剂对照的多中心临床试验。

2. 研究负责单位以及主要研究者的资质情况

The qualifications of the research institution and the main researchers

本临床研究的负责单位——中国中医科学院广安门医院，集医疗、教学、科研和预防保

健为一体的三级甲等中医医院。

主要研究者魏军平，主任医师，教授。长期从事中西医结合防治糖尿病及其慢性并发症等内分泌代谢疾病的临床、科研、教育。

3. 哪些人将被邀请参加这项研究 ？

Who will be invited to participate in the trial?

▲纳入本研究的条件包括 (inclusion criteria):

（1）符合西医 2 型糖尿病、糖尿病肾病诊断标准；

（2）符合中医辨证气阴两虚兼夹血瘀证诊断标准；

（3）UACR≥30 mg/g；

（4）eGFR≥30 ml/min/1.73 m^2 且≤59 ml/min/1.73 m^2（CKD-EPI 公式计算）；

（5）空腹血糖<13.9 mmol/L 和（或）餐后 2 小时血糖<16.6 mmol/L；

（6）HbA1c≤10%；

（7）年龄≥35 岁且≤75 岁，性别不限；

（8）自愿签署知情同意书。

▲若您具备以下任一项，将不能参与本研究

(If you meet any of the following criteria, you will not be able to participate in this trial):

（1）1 型糖尿病；

（2）单纯肾源性血尿或蛋白尿伴血尿，或肾功能正常但突然出现水肿和大量蛋白尿，显著肾小管功能减退，合并明显的异常肾小管型，有原发性肾小球肾炎或除糖尿病肾病之外的继发性肾炎，泌尿系统急、慢性感染；

（3）筛选时服用氯沙坦钾片的剂量>50 mg/日者；

（4）血红蛋白<90 g/L；

（5）血清白蛋白<30 g/L；

（6）肾动脉狭窄；

（7）血钾＞5.5 mmol/L；

（8）血肌酐≥3 mg/dL（256μmol/L）；

（9）三个月内发生过严重心血管疾病：有心梗病史，或进行过冠脉搭桥或 PCI 手术的缺血性心脏病、充血性心衰（心功能III-Ⅳ级，NYHA 评级）患者，或有严重脑血管疾病患者（卒中及卒中后遗留明显神经系统功能障碍）；

（10）合并肝、造血系统、内分泌系统、呼吸系统等其他严重全身性原发性疾病及功能障碍患者；ALT、AST＞正常值上限 2.5 倍者；

（11）服用 3 种以上降压药物者，或服用降压药后仍控制不良的高血压，收缩压≥140

mmHg，舒张压≥90 mmHg；或有低血压（静息坐位血压）收缩压<90 mmHg，舒张压<60 mmHg 的患者；

（12）慢性腹泻者，定义为排便次数增多（>3 次/日），粪便量增加，粪质稀薄，腹泻超过 3~6 周或反复发作；

（13）三个月内曾发生活动性出血者；

（14）凝血功能异常者；

（15）过敏体质、对多种药物过敏或对试验药物或氯沙坦钾片成分过敏者；

（16）有酗酒、滥用药物或精神疾病史者；

（17）处于妊娠期、哺乳期或计划在研究期间妊娠的女性；

（18）近 3 个月内参加过其他临床试验者；

（19）研究者认为其他不适宜参加本临床试验者。

4. 有多少人参加这项研究？

How many participants will be enrolled in this trial?

该项临床研究预计全国将有 17 家医院参加，拟入组 252 例受试者。

5. 该研究的研究过程是怎样的？

What is the main content and the procedure of this trial?

如果符合纳入受试者的条件，您将进入为期 24 周的治疗周期，您将随机分入试验组和对照组，您有 50%的概率进入试验组，有 50%的概率进入对照组。

试验组服用渴络欣胶囊，一次 4 粒，一日 3 次；同时服用氯沙坦钾片，50 mg/片，1 片/次，1 次/日。对照组服用渴络欣胶囊模拟剂，一次 4 粒，一日 3 次；同时服用氯沙坦钾片，50 mg/片，1 片/次，1 次/日。

渴络欣胶囊和渴络欣胶囊模拟剂在外观如颜色、性状、气味等方面没有肉眼可见的差别，但是渴络欣胶囊模拟剂不含药物有效成分，服用渴络欣胶囊模拟剂可能没有相应的治疗作用。

您和您的研究医生都不能选择您的治疗组别，在研究期间，您和您的医生在试验中不会知道您所处的组别。

整个研究过程中，在我们将通过一系列的检查收集您对研究药物的反应和您的健康状况。

（1）筛选期:V0（第-28~-15 天）

- 采集一般资料：您自愿签署知情同意书参加该项研究后，医生将询问您的基本情况、既往病史及治疗用药情况、过敏史、现病史等，对您进行下列检查。
- 筛选检查：育龄期女性需进行尿妊娠试验；空腹血糖和（或）餐后 2 小时血糖；肾脏彩超及肾动脉彩超。

- 生命体征检查：脉搏、血压、呼吸、体温。
- 实验室检查：包括血常规（RBC、Hb、WBC、PLT），尿常规及尿沉渣镜检，24 h 尿蛋白定量（UTP），尿白蛋白/肌酐比（UACR），肾功能（Scr，eGFR），肝功能（ALT、AST、AKP、TBIL、GGT、ALB），电解质（K^+、Na^+、Cl^-），血脂（TC、TG、HDL-C、LDL-C），凝血功能（PT、APTT、FIB），糖化血红蛋白（HbA1c）。
- 特殊检查：十二导联心电图。
- 进行纳入标准和排除标准的检查。
- 根据您筛选时是否服用氯沙坦钾片，分别进行以下检查：

  ① 如果您正在筛选时服用氯沙坦钾片（50 mg/日），无需进行氯沙坦钾片的剂量滴定，若您符合纳入标准且不符合排除标准，即可随机入组，进入第 0 天基线期（V2）。

  ② 如果您筛选时未服用过氯沙坦钾片，筛选期检查结果提示您符合纳入标准且不符合排除标准，则需要进入滴定期（V1），研究者为您发放氯沙坦钾片，进行氯沙坦钾片的剂量滴定（详见滴定期：V1）。
- 研究医生将会记录您在筛选期的不适以及合并用药情况。

（2）滴定期:V1（第-14~ -1 天）

① 如果您在筛选时每天服用 1 片 50 mg 氯沙坦钾片：

您无需进行氯沙坦钾片滴定，直接进入第 0 天基线期（V2）进行中医证候评分。

② 如果您筛选时未服用过氯沙坦钾片，则需要进行氯沙坦钾片剂量滴定试验：

您将口服氯沙坦钾片，50 mg/片，1 片/次，1 次/日，连续口服 2 周，服药期间每日自测血压 4 次（注：滴定期服用氯沙坦钾片≥12 天且≤18 天均符合要求）。

- **2 周的滴定期内如您出现以下低血压的情况，您将不能入组：**

经调整除氯沙坦钾片（50 mg/日）外的其它降压药后，任一次的平均血压符合低血压标准：SBP＜90 mmHg 且 DBP＜60 mmHg（静息状态下坐位测量上臂血压，单次血压重复测量 2 次后取平均值），则不能入组。您自己测量到低血压后，请告知研究者，并携带剩余研究药物尽快回到医院，由研究者为您进行相应的医学判断和处理。

（3）基线期：V2（第 0 天）

① 如果您在筛选时每天服用 1 片 50 mg 氯沙坦钾片：

您将接受生命体征检查：脉搏、血压、呼吸、体温，完成中医证候疗效评价。研究医生将记录您的不适和合并用药的情况，为您发放研究药物。

② 如果您筛选时未服用过氯沙坦钾片并接受氯沙坦钾片剂量滴定，您将接受以下检查：

- 生命体征检查：脉搏、血压、呼吸、体温。
- 实验室检查：肾功能（Scr，eGFR）、电解质（K^+、Na^+、Cl^-）、血常规（RBC、Hb、

WBC、PLT)、尿常规及尿沉渣镜检、24 h 尿蛋白定量(UTP)、尿白蛋白/肌酐比(UACR)、空腹血糖和（或）餐后 2 小时血糖。

- 进行氯沙坦钾片剂量滴定耐受结果判断：

如出现以下任意 1 项，您将不能入组：

- 经调整除氯沙坦钾片（50 mg/日）外的其它降压药后，SBP＜90 mmHg，DBP＜60 mmHg（静息状态下坐位测量上臂血压，单次血压重复测量 2 次后取平均值)；
- 血肌酐增加＞基线 30%；
- 血钾>5.5 mmol/L；
- eGFR<30 ml/min/1.73 m^2 或>59 ml/min/1.73 m^2。

- 如您能够耐受氯沙坦钾片（50 mg/日），则需再次核对纳入标准和排除标准。
- 如您符合纳入标准且不符合排除标准，则随机入组。
- 疗效观察：中医证候评分。
- 研究医生记录不良事件、合并用药，回收滴定试验中发放的氯沙坦钾片，发放治疗期所使用研究药物。

（4）治疗期：V3（第 4 周±3 天）

- 生命体征检查：脉搏、血压、呼吸、体温。
- 实验室检查：
  - 血常规（RBC、Hb、WBC、PLT)；
  - 尿常规及尿沉渣镜检；
  - 24 h 尿蛋白定量（UTP)；
  - 尿白蛋白/肌酐比（UACR)；
  - 肾功能（Scr，eGFR)；
  - 电解质（K^+、Na^+、Cl^-)；
  - 凝血功能（PT、APTT、FIB)；
  - 空腹血糖。
- 疗效观察
  - 血肌酐加倍发生率；
  - ESRD 发生率；
  - 24 h 尿蛋白定量（UTP）较基线变化的百分比；
  - 尿白蛋白/肌酐比（UACR）较基线的变化；
  - 中医证候评分。

- 记录不良事件、合并用药，发放研究药物，回收剩余研究药物。

（5）治疗期：V4（第 12 周±5 天）

- 生命体征检查：脉搏、血压、呼吸、体温。
- 实验室检查：
  - 血常规（RBC、Hb、WBC、PLT）；
  - 尿常规及尿沉渣镜检；
  - 24 h 尿蛋白定量（UTP）；
  - 尿白蛋白/肌酐比（UACR）；
  - 肝功能（ALT、AST、AKP、TBIL、GGT、ALB）；
  - 肾功能（Scr，eGFR）；
  - 电解质（K^{+}、Na^{+}、Cl^{-}）；
  - 血脂（TC、TG、HDL-C、LDL-C）；
  - 凝血功能（PT、APTT、FIB）；
  - 糖化血红蛋白（HbA1c）；
  - 空腹血糖。
- 疗效观察
  - 血肌酐加倍发生率；
  - ESRD 发生率；
  - 24 h 尿蛋白定量（UTP）较基线变化的百分比；
  - 尿白蛋白/肌酐比（UACR）较基线的变化；
  - 中医证候评分。
- 记录不良事件、合并用药，发放研究药物，回收剩余研究药物。

（6）治疗期：V5（第 24 周±5 天）

- 生命体征检查：脉搏、血压、呼吸、体温。
- 实验室检查：
  - 血常规（RBC、Hb、WBC、PLT）；
  - 尿常规及尿沉渣镜检；
  - 24 h 尿蛋白定量（UTP）；
  - 尿白蛋白/肌酐比（UACR）；
  - 肝功能（ALT、AST、AKP、TBIL、GGT、ALB）；
  - 肾功能（Scr，eGFR）；
  - 电解质（K^{+}、Na^{+}、Cl^{-}）；

- 血脂（TC、TG、HDL-C、LDL-C）；
- 凝血功能（PT、APTT、FIB）；
- 糖化血红蛋白（HbA1c）；
- 空腹血糖；

➢ 特殊检查：

- 十二导联心电图

➢ 疗效观察

- eGFR 较基线下降速率（ml/min/1.73 m^2/半年）；
- 血肌酐加倍发生率；
- ESRD 发生率；
- 半年内 eGFR 较基线进行性下降幅度＞30%的受试者比例；
- 24 h 尿蛋白定量（UTP）较基线变化的百分比；
- 尿白蛋白/肌酐比（UACR）较基线的变化；
- 中医证候疗效。

➢ 记录不良事件、合并用药，回收剩余研究药物，依从性判断。

6. 参加试验被终止的预期情况和原因

Expected circumstances and reasons for termination of the trial

如试验过程中，您达到以下任一项，即终止试验：

（1）肌酐翻倍；

（2）进展为 ESRD（eGFR＜15 ml/min/1.73 m^2 或依赖透析超过 3 月）；

（3）受试者死亡。

7. 参加该研究对您日常生活的影响

What are the impacts on your daily life if you participate in the trial?

当您决定是否参加本研究时，请仔细考虑如上所列的检和 5 次访视对您的日常工作、家庭生活等可能的影响。考虑每次访视的时间与给药后身体舒适问题。若您对试验涉及的检查和步骤有任何疑问，可以向我们咨询。

8 参加研究期间的注意事项

Precautions during the study period

8.1 试验期间禁止服用以下药物：

The following drugs are prohibited during the trial

禁止使用试验方案许可以外的治疗或影响糖尿病肾病病情变化的其它药物，包括血管紧张素转换酶抑制剂（ACEI）或除氯沙坦钾片以外的其他血管紧张素Ⅱ受体拮抗剂（ARB）

类药物、羟苯磺酸钙、包醛氧淀粉、药用活性炭等，以及其他影响药物疗效评价的用于气虚血瘀证的中药制剂。

8.2 试验期间允许使用的合并用药

Combination drugs allowed during the trial

（1）降糖治疗：根据受试者血糖和糖化血红蛋白（HbA1c）水平，给予单一口服降糖药、注射胰岛素或联合降糖治疗，使受试者血糖控制稳定；

（2）降压治疗：可联用非肾素-血管紧张素-醛固酮系统抑制剂（RASI）降压药物控制血压达标；如果血压<110/60 mm Hg，必须减少所使用的降压药物种类；

（3）降脂治疗：根据受试者血脂水平及心血管风险使用他汀类降脂药物；

（4）饮食治疗：蛋白质摄入0.6- 0.8 g/kg/d，可补充复方α--酮酸制剂0.12 g/kg/d；钠盐摄入＜3 g/d（相当于氯化钠6 g/d)）；

（5）CKD的其它治疗：纠正高尿酸血症（采用别嘌呤醇，非布司他等）、贫血（可给予重组人红细胞生成素和铁剂等治疗），治疗慢性肾脏病矿物质和骨异常（CKD-MBD）等并发症；

（6）血肌酐升高≥基线50%（连续2次），评价是否合并低血容量、药物、感染，低心排出量、尿路梗阻等情况，停用可疑药物。4周后复查血肌酐，如恢复则继续服用试验药物；如未恢复至基线50%以内或持续升高或进展为ESRD，则达到试验终点；

（7）如血钾持续大于5.5 mmol/L，在详细询问受试者饮食、尿量情况下可通过调整饮食、给予袢利尿剂治疗，如仍为高血钾，停用试验药物，退出试验。

9. 参加本研究可能的获益

The possible benefits of joining the trial

通过参加本项临床研究，您的病情有可能获得改善，但不能保证一定改善您的健康状况。您对本研究做出的贡献也有可能使与您病情相同的其他患者获益。在研究过程中出现任何本研究的新信息，医生都将及时告知您。您将在研究期间获得良好的医疗服务。

10. 参加本研究过程中使用的研究药物和相关检查

Study drugs and examination during the trial

本研究期间，您所使用的研究药物（包括试验药物和基础用药）都由申办方提供，并且申办方将为您支付与本次临床试验相关的各项医学检查费用，包括血常规、尿常规及尿沉渣镜检、24 h 尿蛋白定量、尿白蛋白/肌酐比、肝功能、肾功能、电解质、血脂、凝血功能、糖化血红蛋白、空腹血糖和（或）餐后 2 小时血糖、尿妊娠（仅育龄期妇女）、十二导联心电图、肾脏彩超及肾动脉彩超。

11. 参加本研究可能的风险和不良反应

Possible risks and potential adverse reactions resulting from participation in this trial

所有治疗药物都有可能产生副作用。不同人群、生活习惯、饮食习惯等不同，都可能会对用药的治疗效果造成影响。临床治疗中的抽血检查存在一定的风险，如短暂的疼痛、局部青紫，少数人会有轻度头晕，或极为罕见的针头感染等。

另外服用本研究中的试验用药可能会出现以下不良反应：

渴络欣胶囊不良反应：个别患者偶见腹痛、腹泻。

氯沙坦钾片不良反应：上市后已报告的不良反应包括：

（1）过敏反应：血管性水肿(包括导致气道阻塞的喉及声门肿胀，及/或面、唇、咽和/或舌肿胀)在极少数服用氯沙坦治疗的病人中有报道。其中部分病人以前曾因服用包括 ACE 抑制剂在内的其他药物而发生过血管性水肿。脉管炎，包括亨诺克-舍恩莱因(亨-舍二氏)紫癜已有极少报道。

（2）胃肠道反应：肝炎(少有报道)，肝功能异常。

（3）血液系统：贫血。

（4）肌肉骨骼系统：肌痛。

（5）神经/精神系统：偏头痛，癫痫大发作。

（6）呼吸系统：咳嗽。

（7）皮肤：荨麻疹，瘙痒。

（8）高钾血症和低钠血症已有报道。

（9）国内有一例与药物有关的不明原因死亡的自发报告。

（10）实验室检查结果：在原发性高血压临床对照试验中，很少有应用本品的患者在实验室参数方面出现临床上有重要意义的变化。1.5%的患者出现高血钾症（血清钾＞5.5 mEq/L）。在 2 型糖尿病伴蛋白尿的患者中进行的一项临床研究中，氯沙坦组和安慰剂分别有 9.9%和 3.4%的患者出现高钾血症。

（11）ALT 的升高较罕见，并在停药后恢复正常。

（12）肌酐、尿素氮：在原发性高血压患者中，单独使用本品有不到 0.1%的患者观察到血尿素氮或血清肌酐轻微升高。

（13）血红蛋白和红细胞压积：在单独用本品治疗的患者中经常出现血红蛋白和红细胞压积下降（分别平均下降约 0.11%和 0.09%），但很少有临床重要性，没有患者因为贫血而中止服药。

（14）肝功能检查：偶尔有肝脏酶和/或血清胆红素升高。在单独用本品治疗的原发性高血压患者中，一个患者由于这些实验室不良反应而停止服药。

如果在研究中您出现任何不适，或病情发生新的变化，或任何意外情况，不管是否与药

物有关，均应及时通知您的医生，医生将对此做出判断和医疗处理。

如果医生发现本项研究所采取的治疗措施无效，将会中止研究，改用其它可能有效的治疗措施。

12. 发生研究相关伤害时如何处理？

Compensation to those who suffer harm from trial participation

研究医生和申办者（成都康弘制药有限公司）将尽力预防由于本研究可能带来的伤害。如果在临床试验中出现不良事件，为了您的健康，请您立即告知医生。医生会对您展开积极治疗或转诊。若经医生或专家组认定不良事件确与本研究有关，将根据您的损伤情况，申办者将对与试验相关的损害提供治疗的费用及相应的经济补偿，这一点已经在我国《药物临床试验质量管理规范》中做出了规定。

13. 是否参加并完成本研究是您的自愿选择

Trial participation and completion are voluntary

您参加本研究完全是自愿的，您可以选择不参加本项研究，这对您获得常规治疗不会带来任何不良影响。研究医生将会针对您的情况选用合适的治疗方案。

此外，您有权在研究的任何阶段退出研究，如果您决定不参加本研究和研究开始后的任何时间退出本研究，请与您的医生联系，并且退出研究不会受到任何惩罚或利益损失，也不会影响您与医生的关系，医生将根据您的病情对您进行合理的治疗。

出现下列情况时，您的医生可以未经您同意而终止本研究：

（1）出现严重不良事件，

（2）研究过程中出现严重的合并疾病，

（3）违反研究方案，未能遵循研究的有关规定，未按医生指导按时用药或未按时进行各项检查。

14. 受试者可以选择哪些其他的治疗药物？

Are there other treatment drugs that could substitute for the research product?

如您不参加本项临床研究，可以考虑选择的其他治疗药物有：

（1）培哚普利：起始剂量 2 mg/d，最大剂量不超过 8 mg/d；

（2）厄贝沙坦：初始剂量 75 mg/d，逐渐增加至 150 mg/d，最大不超过 300 mg/d；

上述药物仅列出部分可考虑选择的主要治疗药物，您的主治医生将根据您的病情确定选用何种药物以及相应的治疗方案。

15. 受试者的个人信息是否得到保密？

Will your personal information be kept confidential?

一切有关您的信息，包括您的身份、医疗史、病情、体检及实验室检查结果等，都将在法律允许的范围内得到严格保密。研究者、申办方委派的监查员、伦理委员会、及国家食品药品监督管理部门被允许查阅您的与本研究相关的医疗记录，以证实本研究所收集资料的真实性和准确性，但不涉及您的个人详细资料。您的姓名不会出现在任何与此项研究相关的公开资料或报告中。

16. 发现研究药物预期以外的临床影响时会修改并重新签署知情同意书

Will the informed consent form be revised and resigned when unintended clinical effects are detected during the trial?

当发现研究药物预期以外的临床影响时，研究医生在必要的情况下将对知情同意书的相关内容进行修改，并经您或您的法定代理人重新签名确认。

17. 如何获取更多信息，跟谁联系？

Who can I contact if I have further questions?

您可以随时向您的研究医生了解和咨询本研究的任何问题。您的研究医生姓名和电话请见知情同意签字页。另若本品或同类产品在国内外研究过程中获得新的进展、结论或重要事件时，研究者均会及时告知您。如果您有与本研究相关的任何问题，可与您的负责医生联系（联系方式见下页），如果您有与受试者自身权益相关的问题，可以与医院的伦理委员会联系，伦理委员会电话：010-88001552。

知情同意书•同意签字页

第二部分 受试者知情同意 (part 2 Informed consent)

受试者声明(Statement of participants)：

我已阅读并理解“渴络欣胶囊联合氯沙坦钾治疗糖尿病肾病（气阴两虚兼夹血瘀证）随机、双盲、安慰剂对照、多中心临床研究”受试者知情同意书，并已充分理解了该临床研究目的、内容、方法以及可能的获益和风险。医师已将相关的医疗术语做了明确的解释，我所询问的所有问题均得到了通俗易懂的解答。我理解我可以拒绝加入研究或在任何时候和情况下中止和退出研究，医疗待遇和权利不受影响。

我参加本研究完全是自愿的，并有充足时间进行充分考虑，已了解研究药物会对本人疾病带来的治疗作用和可能发生的风险，获得了与本研究有关的完整、真实的信息，本人充分理解和支持该项临床研究。在没有任何压力且能自由选择的情况下，我自愿参加本临床研究，并自愿和研究医师配合，按规定用药、接受体检，完成此项临床研究。

我同意在需要的时候由国家食品药品监督管理部门核查人员、临床研究稽查和监查人员查阅我的病历研究资料。我将获得一份经过签名并注明日期的知情同意书副本。

受试者姓名(participant' s name)（正楷）：______ 受试者签名(signature of participant)：_____

联系方式（电话 tel）：_____________________

受试者法定代理人姓名（正楷）：_________________法定代理人签名：__________________

(Name of the participant' s legal representative) (signature of the participant's legal representative)

法定代理人与受试者关系(relationship with the participant)：______联系方式（电话 tel）：____

签字日期(date)：20|_||_|年/|_||_|月/|_||_|日

研究者声明 (Statement of investigator)：

我已向参加“渴络欣胶囊联合氯沙坦钾治疗糖尿病肾病（气阴两虚兼夹血瘀证）随机、双盲、安慰剂对照、多中心临床研究”的受试者详细解释了本研究的内容、步骤、包括可能的获益和风险。并就受试者提出的任何问题给予了充分的解答，受试者已得到满意的答复并表示理解。我会密切关注、记录临床研究中发生的不良事件、药物的不良反应，并积极采取适当的治疗措施。保证受试者随时能和我取得联系。我将给受试者一份已经签署并注明日期的知情同意书副本。

研究者姓名(investigator's name)（正楷）：_____研究者签名(signature of investigator)：_______

联系方式（电话 tel）：____________________

签字日期(date)：20|_||_|年/|_||_|月/|_||_|日
